# Discovery of a Sexual Cycle in Aspergillus lentulus, a Close Relative of A. fumigatus

**DOI:** 10.1128/EC.00040-13

**Published:** 2013-07

**Authors:** Sameira S. Swilaiman, Céline M. O'Gorman, S. Arunmozhi Balajee, Paul S. Dyer

**Affiliations:** School of Biology, University of Nottingham, University Park, Nottingham, United Kingdoma; Center for Global Health, Centers for Disease Control and Prevention, Atlanta, Georgia, USAb

## Abstract

Aspergillus lentulus was described in 2005 as a new species within the A. fumigatus sensu lato complex. It is an opportunistic human pathogen causing invasive aspergillosis with high mortality rates, and it has been isolated from clinical and environmental sources. The species is morphologically nearly identical to A. fumigatus sensu stricto, and this similarity has resulted in their frequent misidentification. Comparative studies show that A. lentulus has some distinguishing growth features and decreased *in vitro* susceptibility to several antifungal agents, including amphotericin B and caspofungin. Similar to the once-presumed-asexual A. fumigatus, it has only been known to reproduce mitotically. However, we now show that A. lentulus has a heterothallic sexual breeding system. A PCR-based mating-type diagnostic detected isolates of either the *MAT1-1* or *MAT1-2* genotype, and examination of 26 worldwide clinical and environmental isolates revealed similar ratios of the two mating types (38% versus 62%, respectively). *MAT1-1* and *MAT1-2* idiomorph regions were analyzed, revealing the presence of characteristic alpha and high-mobility-group (HMG) domain genes, together with other more unusual features such as a *MAT1-2-4* gene. We then demonstrated that A. lentulus possesses a functional sexual cycle with mature cleistothecia, containing heat-resistant ascospores, being produced after 3 weeks of incubation. Recombination was confirmed using molecular markers. However, isolates of A. lentulus failed to cross with highly fertile strains of A. fumigatus, demonstrating reproductive isolation between these sibling species. The discovery of the A. lentulus sexual stage has significant implications for the management of drug resistance and control of invasive aspergillosis associated with this emerging fungal pathogen.

## INTRODUCTION

Aspergillus lentulus is an opportunistic human pathogenic fungus from the Aspergillus section Fumigati (1) that can cause invasive aspergillosis in immunocompromised patients (2). It was first discovered in 2004, as an unexpected result from a U.S. study examining the susceptibility of isolates of A. fumigatus to azole antifungal drugs (3). Four A. lentulus isolates that caused fatal infections in hematopoietic stem cell transplant patients between the years 1995 and 2000 had been misidentified as poorly sporulating variants of A. fumigatus because their 18S rRNA sequences matched that of A. fumigatus. However, all of the isolates had distinct random amplified polymorphic DNA (RAPD) fingerprint patterns and mitochondrial cytochrome *b* sequences, and several exhibited *in vitro* antifungal susceptibility profiles with significantly higher resistance than normal for A. fumigatus. A follow-up study examined the phylogenetic relationships of these isolates to A. fumigatus using multilocus sequence typing (2). The analysis revealed that the four isolates formed a separate clade clearly distant from A. fumigatus, and it was concluded that these represented a new species, which was named A. lentulus (2). A subsequent phylogenetic study of Japanese clinical isolates showed that along with A. fumisynnematus, A. lentulus forms a sister clade to A. fumigatus (4).

Similar to other members of the section Fumigati that are closely related to A. fumigatus, such as A. fumigatiaffinis, many of the phenotypic characters of A. lentulus overlap with A. fumigatus. This makes identification difficult when based solely on morphological grounds, which often leads to misdiagnoses in clinical laboratories (5). However, a variety of methods have since been found that can differentiate between the two species. Phenotypic dissimilarities include their conidial ornamentation (4), conidiophore architecture, growth characteristics (1), and mycotoxin profiles—most notably the inability of A. lentulus to produce gliotoxin (1, 6). The most obvious growth characteristics that distinguish A. lentulus from A. fumigatus are its delayed onset of sporulation and inability to grow at 48°C, although it should be noted that these two features are common to several other species in the section Fumigati (7). Species-specific molecular methods are also available to identify A. lentulus and include the use of a microsphere-based Luminex assay (8), RAPD patterns (1), a multiplex PCR assay (9), and restriction fragment length polymorphisms (10).

Despite the wide geographic distribution and presence of the species in common environmental niches (3–5, 7, 11–13), opportunistic A. lentulus infections appear rare. To date, only six studies have reported cases of invasive aspergillosis in which A. lentulus was confirmed as the probable or causal agent (3, 11, 12, 14–16). Two further cases involved the colonization of cystic fibrosis patients (13, 17). This apparent low incidence rate may be due to the misidentification problems described previously (5), because A. lentulus isolates have been recovered in several retrospective studies of clinical fungal samples (4, 7).

The pathology of invasive infections caused by A. lentulus appears to mirror that of A. fumigatus (3). However, of medical significance is the fact that most isolates exhibit an increased natural resistance to several antifungal agents currently in clinical use compared to A. fumigatus, namely, itraconazole, voriconazole, caspofungin, and amphotericin B (3, 4, 7). The molecular mechanisms underlying these resistance mechanisms have recently started to be elucidated (18, 19), but further work is required to fully understand the genetic basis of resistance. Of particular interest are the mechanisms governing echinocandin resistance, as A. lentulus is highly unusual in being simultaneously resistant to caspofungin yet highly sensitive to anidulafungin and micafungin (7, 19, 20). The discovery of A. lentulus has also proven beneficial to industry, as an A. lentulus isolate has been found with activity as a biosorbent for the removal of toxic compounds. A. lentulus strain AML05, which was recovered from industrial textile effluent in India by a chromium enrichment process, can very successfully remove Cr(VI) from electroplating industry effluent (21, 22) and dyes from textile effluent (23, 24).

A. lentulus is currently known to reproduce only by asexual means, through the production of conidia (2). Hong and coworkers (1) previously tried unsuccessfully to mate strains of A. lentulus, but crucially, this was before recent reports of the discovery of a sexual cycle in A. fumigatus (teleomorph Neosartorya fumigata) (25) and other related Aspergillus and Penicillium species which were previously considered asexual (26–31). In most of these cases, the discovery of a sexual state was preceded by the identification of mating-type (*MAT*) genes within the species, such genes acting as key regulators of sexual identity in filamentous ascomycete fungi (32, 33). In heterothallic (obligate outbreeding) species, these *MAT* genes are contained within a region of the genome termed the *MAT* locus, with highly divergent forms of this locus, known as “idiomorphs,” present in isolates of sexually compatible *MAT1-1* and *MAT1-2* genotypes. In contrast, homothallic (self-fertile) species may contain one or more *MAT* loci within the same genome rather than dissimilar idiomorphs (33, 34).

The close phylogenetic relationship of A. lentulus to A. fumigatus suggested that it might be possible to induce sexual reproduction in A. lentulus using conditions similar to those required for A. fumigatus. The aims of this study were, therefore, first, to investigate the presence of *MAT* genes in A. lentulus to see if isolates with putative sexual compatibility could be identified; second, to determine if a sexual cycle could be induced in A. lentulus; third, to use molecular analysis of offspring to determine if the breeding system was heterothallic or homothallic in nature; and, finally, to see if any gene flow was possible between A. lentulus and A. fumigatus via sexual crossing. The ability to perform sexual crosses in A. lentulus would provide a valuable tool for the genetic analysis of traits relating to pathogenicity, antifungal drug resistance, and industrial processes in this species.

## MATERIALS AND METHODS

### Strains, growth conditions, and DNA extraction.

Twenty-six isolates of A. lentulus from clinical and environmental sources were used in the study, comprising most isolates of the species previously reported in the scientific literature (see Table S1 in the supplemental material). Sixteen of the clinical isolates were kindly donated by Kieren Marr and Edmond Byrnes (John Hopkins School of Medicine, Maryland). Strains were maintained on Aspergillus complete medium (ACM) (35) at 28°C and are stored in 10% glycerol under liquid nitrogen at the School of Biology, University of Nottingham, United Kingdom (BDUN [Botany Department, University of Nottingham] culture collection). Genomic DNA was extracted using a DNeasy plant minikit (Qiagen) in accordance with the manufacturer's instructions. Cultures were grown in liquid ACM at 28°C for 5 days. The resulting mycelia were harvested, flash frozen, and ground under liquid nitrogen prior to DNA extraction.

### Multiplex mating-type PCR assay and PCR amplification of the mating-type idiomorphs.

The mating-type genotype of all A. lentulus isolates and ascospore progeny was determined with the A. fumigatus multiplex mating-type PCR diagnostic of Paoletti et al. (35), using primers AFM1, AFM2, and AFM3 (see Table S2 in the supplemental material). Attempts were then made to amplify the entire *MAT* idiomorph regions of A. lentulus isolates 78-2 and 78-3 with the primer pair Loc1 and AFM3 (see Table S2) using the conditions described by Paoletti et al. (35); these primers had previously been used to successfully amplify the idiomorph regions of A. fumigatus (35). Resulting amplicons were purified using a Geneflow Q-Spin PCR purification kit (according to the manufacturer's instructions) and were sequenced using primers AL31-AL34 and AL51-AL53 (see Table S2) at the DNA Sequencing Facility of the School of Biomedical Sciences, University of Nottingham, United Kingdom. Arising sequences were analyzed and aligned using MacVector 11 (MacVector Inc.).

### Sexual crosses.

Sixteen representative A. lentulus isolates of *MAT1-1* and *MAT1-2* genotype (see Table S1 in the supplemental material) were chosen for sexual crossing experiments and inoculated in all possible pairwise combinations by following the protocol of O'Gorman et al. (25). Briefly, crosses were set up on oatmeal agar (pinhead oatmeal, Odlums, Ireland [36]) in triplicate, sealed with one layer of Nescofilm, and incubated at 25, 28, or 30°C in the dark. Four A. fumigatus crosses known to reliably produce cleistothecia and ascospores (AfRB2 × AfIR928, AfRB2 × AfIR964, AfIR974 × AfIR928, and AfIR974 × AfIR964) (25) were set up in parallel as controls. Control “selfed” A. lentulus crosses were also tested on oatmeal agar at 28°C using one representative isolate of each mating type (78-2 [*MAT1-2*] and 78-3 [*MAT1-1*]). In addition, crosses were set up on oatmeal agar at 28°C and 30°C between representative A. lentulus *MAT1-1* (78-3, 78-6, and 78-20) and *MAT1-2* (78-2 and 78-8) isolates (see Table S1 in the supplemental material) and known highly fertile isolates of A. fumigatus (AFB62 [*MAT1-1*] and AfIR928 [*MAT1-2*]) (37) to assess reproductive isolation. All crosses were examined periodically for the presence of cleistothecia for up to 7 weeks using a Nikon-SMZ-2B dissection microscope and then, finally, after 4 and 12 months of incubation where cleistothecia were not detected in the initial growth period.

### Preparation of single-ascospore cultures.

Mature cleistothecia from the cross 78-2 × 78-3 were removed and cleaned as described previously (25), with the modification that 4% water agar was used to clean the cleistothecia instead of a drop of sterile water. Five cleistothecia were added to 50 μl of 0.05% Tween 80 (BDH Chemicals) under sterile conditions and ruptured by squashing with a needle tip. The solution was brought up to 500 μl with 0.05% Tween 80 and vortex mixed for 1 min to release the ascospores. The suspension was then heat treated at 80°C for 30 min, this temperature being sufficient to kill any contaminating conidia without damaging the ascospores (data not shown). One hundred microliters of a 5 × 10^5^-ascospore ml^−1^ heat-treated suspension was spread inoculated on three defined areas of an ACM plate. Triplicates were prepared and incubated at 37°C for 14 h. Single-spore cultures were established on ACM by transferring individual germinating ascospores with a LaRue lens cutter attached to a Nikon-Optiphot microscope.

### Analysis of recombination.

The segregation of five genetic markers (four RAPD bands and the mating-type genotype) in the ascospore progeny was examined for evidence of recombination. RAPD-PCR fingerprinting was performed by following the protocol of O'Gorman et al. (25). Four primers (OMT1, R108, R151, and OPWO8 [see Table S2 in the supplemental material]) from an initial screen of 12 were found to yield suitable polymorphisms for genotyping.

### SEM.

Cleistothecia were collected from 6-month-old crosses of 78-2 × 78-3 that had been incubated at 28°C. Representative intact and crushed cleistothecia were transferred onto 0.2-μm filter discs (Whatman) and fixed in 2% osmium tetroxide (Sigma) for 2 h at room temperature. The fixed samples were then mounted onto aluminum stubs, dried at 37°C overnight, and sputter coated with gold. Scanning electron microscopy (SEM) micrographs were taken using a JSM-840 JEOL scanning electron microscope.

### Statistical analysis.

The hypothesis of a 1:1 ratio of mating types in the worldwide sample population and ascospore progeny was tested using χ^2^ and contingency χ^2^ tests. Where expected frequencies were <5, Fisher's exact test was used instead (38).

### Accession numbers.

The sequences for the two mating-type loci amplified from isolates 78-3 (*MAT1-1*) and 78-2 (*MAT1-2*) have been deposited in GenBank under accession numbers KC876046 and KC876047. The diagnosis of the Aspergillus lentulus (neosartorya-morph) has been deposited in MycoBank under accession number MB356679 (see the supplemental material).

## RESULTS AND DISCUSSION

Sex is thought to have evolved in early eukaryotic microbes and is now widespread throughout the Eukaryota (39). The ability to undergo sexual reproduction is considered to be of major importance given the many benefits it confers. These include the potential to purge harmful mutations and improve the fitness of offspring, which, in turns, allows them to better resist adverse environmental conditions (40–43). It is therefore surprising that the kingdom Fungi appears to contain a disproportionally large number of supposedly asexual species, with an estimated 20% having no known sexual stage. Many are members of the phylum Ascomycota that are of medical or economic significance (44). Some, such as A. oryzae, have been shown to possess *MAT* genes and other “sexual machinery,” yet their sexual cycle remains elusive (45). While there are clear advantages to asexual over sexual reproduction, such as the relatively lower metabolic cost and ability to produce spores under a wider range of environmental conditions, the rewards of sexual reproduction appear to be much greater (46).

The discovery of a heterothallic sexual cycle in A. fumigatus, which had long been considered to be reproduce purely by mitotic means, confirmed suspicions that at least some of these supposed “asexuals” do in fact have the potential to reproduce sexually (25, 43). Significantly, a “sexual revolution” has since followed, with the reporting of functional sexual cycles in several other related filamentous fungi (26–31, 47). The aim of this work was to determine whether it was possible to induce a sexual cycle in A. lentulus, given its close phylogenetic relationship to A. fumigatus (1) and its increasing importance as a human pathogen with resistance to drugs in several antifungal classes relative to A. fumigatus (2).

### Presence, distribution, and characterization of the *MAT* idiomorphs.

The A. lentulus genome was first examined for the presence of the master regulatory *MAT* genes that are transcription factors common to all heterothallic fungi and which determine cell sexual identity (32). It was found that the previously described multiplex PCR mating type diagnostic for A. fumigatus (35) produced corresponding amplicons of the predicted size (ca. 834 bp for *MAT1-1* and 438 bp for *MAT1-2*) in different isolates of A. lentulus (see Fig. S1 in the supplemental material). This indicated a heterothallic (obligate outcrossing) arrangement in the species and confirmed the phylogenetic affinity with A. fumigatus, given that this was designed as a species-specific diagnostic test. The worldwide collection of A. lentulus isolates was then screened to determine the ratio of complementary *MAT1-1* and *MAT1-2* genotypes (see Table S1 in the supplemental material). Amplicons were generated for all isolates, and the overall mating-type distribution did not deviate significantly from a 1:1 ratio (38.5% *MAT1-1* and 61.5% *MAT1-2*; χ^2^ = 1.38; *n* = 26), consistent with a sexually reproducing species. When isolates were grouped according to geographic origin, there was also no significant difference in the *MAT* distribution (data not shown).

**Fig 1:**
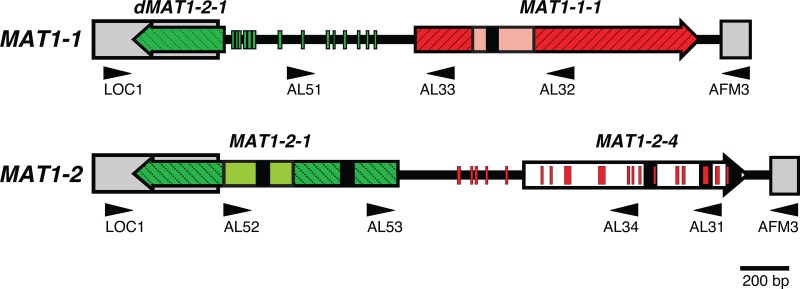
*MAT* locus of Aspergillus lentulus. The arrangement of the A. lentulus idiomorph region shows the difference in organization between isolates 78-3 (*MAT1-1*) and 78-2 (*MAT1-2*). Colored block arrows indicate *MAT1-1-1* (red), *MAT1-2-1* (green), and *MAT1-2-4* (white) sequences. Colored boxes indicate the α1 domain (salmon pink), HMG domain (light green), and nearly identical flanking regions (gray). Introns are represented by black boxes; lines extending between boxes and arrows represent idiomorph sequence. Smaller red and green segments represent regions between 10 and 29 bp in length with ≥70% *MAT1-1-1* and *MAT1-2-1* nucleotide conservation, respectively. Black arrowheads (direction indicates 5′ to 3′ sequence) show the positions of primers (see Table S2 in the supplemental material) used for amplification of the idiomorph region.

It was then found that the entire *MAT1-1* and *MAT1-2* idiomorph regions of A. lentulus could be amplified using the primers Loc1 and AMF3, which had previously been used to amplify *MAT* regions from A. fumigatus (35). This yielded amplicons of 2,524 and 2,731 bp, respectively. Figure 1 shows the complete sequenced idiomorph structure of the two mating-type loci amplified from isolates 78-3 (*MAT1-1*) and 78-2 (*MAT1-2*). The idiomorphs showed the same overall structural organization as those previously reported from A. fumigatus (35). The *MAT1-1* idiomorph of 78-3 contains a 1,157-bp putative open reading frame (ORF), predicted to encode a 368-amino-acid protein with a characteristic α1 domain, and was therefore termed *MAT1-1-1* (33). The ORF is interrupted by a 50-bp intron in the conserved position found in other ascomycetes, and the overall protein and α1 domain region share 93% and 98% amino acid identity to A. fumigatus, respectively (see Fig. S2A in the supplemental material). The *MAT1-2* idiomorph of 78-2 contains two putative ORFs. The first is a 1,077-bp ORF predicted to encode a 322-amino-acid protein with a characteristic high-mobility-group (HMG) box, which was therefore termed *MAT1-2-1* (33). This ORF contains two introns (53 and 55 bp), and the overall protein and HMG domain share 89% and 92% amino acid identity to A. fumigatus, respectively (see Fig. S2B). The second is a putative ORF of 903 bp with three introns (46, 46, and 58 bp), predicted to encode a 242-amino-acid product which was found to share 95% and 87% amino acid identity to the putative MAT1-2-4 proteins from A. fumigatus and Neosartorya fischeri, respectively (see Fig. S2C) (48, 49; C. Eagle and P. S. Dyer, unpublished data). A related *MAT1-2-4* family gene has also been described for Talaromyces (Penicillium) marneffei (50). However, similar *MAT1-2-4* genes are absent from many other aspergilli, and their expression and possible functionality remain to be determined. A *MAT1-2-4* gene has also been reported for Coccidioides immitis and C. posadasii (51, 52), but this shares little sequence conservation with the MAT1-2-4 proteins from the aspergilli and is absent from the *MAT* loci of many other heterothallic eurotiomycete species, such as those recently described for Blastomyces dermatitidis (53).

Similar to the previous report for A. fumigatus (35), although the *MAT1-2-1* gene commenced within the *MAT1-2* idiomorph, a terminal 374-bp region was found to lie within the flanking sequence bordering both idiomorphs (Fig. 1). However, the fragment bordering the *MAT1-1* idiomorph appeared nonfunctional, as it lacked the HMG domain region and any start codon and contained a deletion, giving rise to a frameshift mutation. Therefore, the fragment was termed *dMAT1-1-1* in recognition of the disabled ORF (48). Intriguingly, further analysis of the *MAT1-1* idiomorph revealed an additional 14 regions between 10 and 15 bp in length with ≥70% nucleotide conservation compared to the *A. lentulus MAT1-2-1* gene. These extended directly upstream from the 374-bp *dMAT1-2-1* fragment, with seven of the regions present within the predicted HMG domain (Fig. 1). Similarly, further analysis of the *MAT1-2* idiomorph revealed 18 regions between 10 and 29 bp in length within the idiomorph which exhibited ≥70% nucleotide conservation compared to *A. lentulus MAT1-1-1* gene, including 13 within the *MAT1-2-4* gene itself (Fig. 1). These data indicate a complex evolutionary history for the idiomorphs, possibly signifying the presence of an ancestral homothallic *MAT* locus containing both *MAT1-1-1* and *MAT1-2-1* genes, which has since undergone accelerated mutation and evolution in the transition to heterothallism (54). It has been suggested that homothallism might be the ancestral state within the Aspergillus section Fumigati, with the overwhelming majority of teleomorphic Neosartorya species exhibiting homothallic breeding systems (55, 56). It can also be speculated that the *MAT1-2-4* gene, whose origins are obscure, might have arisen from sequence derived from the *MAT1-1-1* gene.

### Sexual crosses.

**Fig 2:**
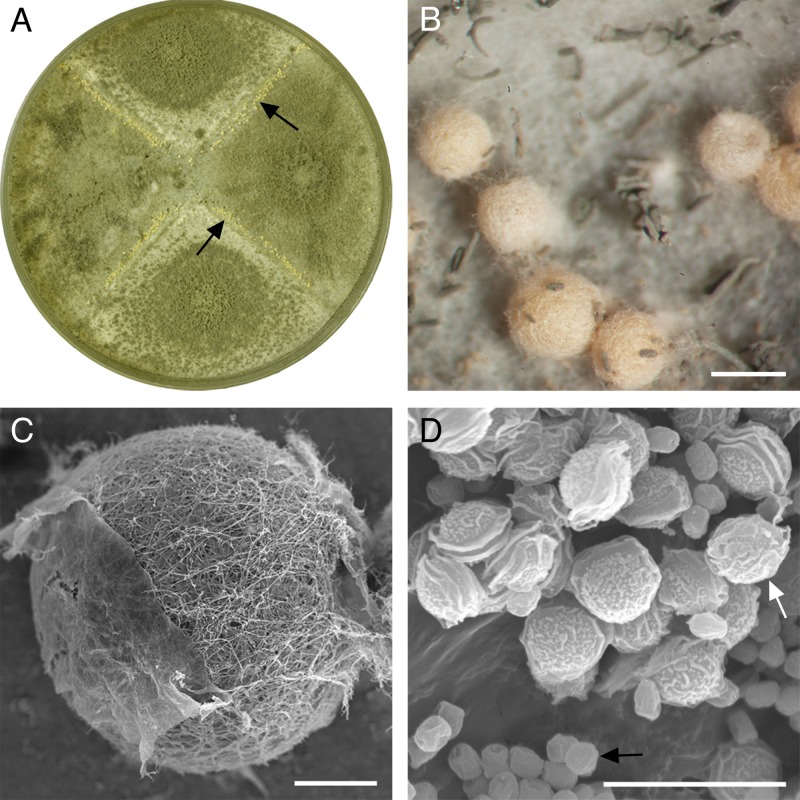
Sexual reproduction in Aspergillus lentulus. (A) Paired cultures of isolates 78-2 (*MAT1-2*) × 78-3 (*MAT1-1*) on oatmeal agar (9-cm-diameter petri dish) with cleistothecia (arrows) along the barrage zones following 3 weeks of incubation at 28°C. (B) Close-up of a barrage zone showing pale yellow cleistothecia. Scale bar, 400 μm. (C) SEM micrograph of a cleistothecium showing the interwoven hyphae that form the peridial wall. Scale bar, 100 μm. (D) SEM micrograph of lenticular ascospores (white arrow) and smaller globose conidia (black arrow). Scale bar, 10 μm.

Crosses were set up between isolates of complementary mating type using the conditions that had successfully induced sex in A. fumigatus (25), with one modification. Both 25°C and 28°C were tested in addition to the 30°C required for A. fumigatus mating, given that A. lentulus has a lower growth temperature range than A. fumigatus (1). Nine isolates of clinical and environmental origin from North America and Asia (see Table S1 in the supplemental material) were crossed in all pairwise combinations (Table 1). Significantly, after 3 weeks of incubation at both 28°C and 30°C, certain crosses were found to be fertile, producing cleistothecia that contained viable ascospores (Table 1 and Fig. 2; see supplemental material for species diagnosis). In contrast, no cleistothecia were observed in crosses incubated at 25°C. Cleistothecia also failed to develop on single *MAT* cultures, indicating that A. lentulus is a heterothallic species.

**Table 1:** Mean numbers of cleistothecia produced by 14 Aspergillus lentulus crosses on oatmeal agar at 28°C or 30°C in the dark after 3 weeks^a^

*MAT1-2 *strain	Score for cleistothecium production of cross with *MAT1-1 *strain			
	28°C		30°C	
	78-3	78-6	78-3	78-6
78-1	−	−	−	−
78-2	>	+	++++	+++
78-4	−	−	−	−
78-5	−	−	−	−
78-7	−	−	−	−
78-8	+	+	+	−
78-9	−	−	−	−

a Ratings indicate the mean number of cleistothecia produced from three replicate crosses on oatmeal agar in 9-cm-diameter petri dishes after incubation in the dark, as follows: −, none; +, 1 to 19; +++, 40 to 59; ++++, 80 to 100; >, more than 100.

**Fig 3:**
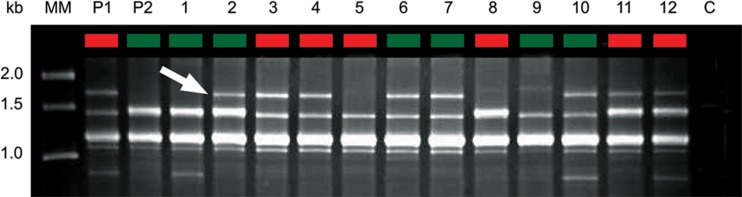
Evidence for meiotic recombination. The gel shows segregation patterns of a RAPD-PCR amplicon in A. lentulus parental isolates (P1 and P2) and 12 ascospore progeny (lanes 1 to 12) from the cross 78-3 (P1) × 78-2 (P2), using primer OPW08. MM, molecular size marker; C, water control. Arrow indicates the diagnostic RAPD band. Red and green lane headings indicate *MAT1-1* and *MAT1-2* genotypes, respectively.

In crosses where cleistothecia were produced, they formed along the barrage zones between isolates of opposite mating type (Fig. 2A) and were pale yellow-orange in color (Fig. 2B). All crosses forming cleistothecia produced viable ascospores. The ascospores were heat resistant (57), capable of surviving at 80°C for at least 30 min, as is typical for other members of the genus Neosartorya (56). This might reflect selection in a common ancestor of Neosartorya for survival in ecological niches where high temperatures might be encountered, such as composting vegetation (58). Ascospore progeny from a cross between isolates 78-2 and 78-3 were then assessed for evidence of recombination. Distinct segregation patterns were clearly observed between four RAPD-PCR markers and the *MAT* genotype in 12 ascospore progeny, with Fisher's exact test confirming 1:1 Mendelian segregation of the markers due to independent assortment, confirming a heterothallic sexual breeding system (Table 2 and Fig. 3). Unique genotypes were found in 92% of the progeny, with only one of the offspring identical to its parent (based on the markers examined).

**Table 2:** Genotypes of parental isolates and 12 ascospore progeny from a cross between A. lentulus isolates 78-2 and 78-3, based on mating type and RAPD-PCR bands

Isolate	Mating type	RAPD band^a^				Genotype^b^
		OMT1	R108	OPW08	R151	
78-3	*MAT1-1*	−	+	+	−	P1
78-2	*MAT1-2*	+	−	−	+	P2
3-2-1	*MAT1-2*	−	−	−	−	A
3-2-2	*MAT1-2*	−	−	+	+	B
3-2-3	*MAT1-1*	−	+	+	−	P1
3-2-4	*MAT1-1*	−	−	+	−	C
3-2-6	*MAT1-1*	−	−	−	−	D
3-2-7	*MAT1-2*	−	−	+	−	E
3-2-8	*MAT1-2*	−	+	+	−	F
3-2-9	*MAT1-1*	−	−	−	+	G
3-2-12	*MAT1-2*	−	+	−	−	H
3-2-13	*MAT1-2*	−	−	+	−	E
3-2-15	*MAT1-1*	+	−	+	−	I
3-2-16	*MAT1-1*	+	−	+	+	J

a RAPD-PCR bands amplified using primers OMT1, R108, OPW08, and R151. “+” and “−” denote presence and absence, respectively, of a particular amplicon. *P* values (two-tailed) for OMT1, R108, OPW08, and R151 were 0.06, 0.24, 0.57, and 0.24, respectively. Fisher's exact test was conducted to check for deviation from the null hypothesis of independent assortment of mating-type and RAPD markers in the progeny (i.e., a 1:1:1:1 *MAT1-1*+:*MAT1-1*−:*MAT1-2*+:*MAT1-2*− ratio for each RAPD marker). Fisher's exact test was used instead of the χ^2^ test because the expected frequencies were <5. A contingency χ^2^ test was conducted to check for deviation from the null hypothesis of independent assortment of mating-type and RAPD markers in the progeny (i.e., an overall 1:1:1:1 *MAT1-1*+:*MAT1-1−*:*MAT1-2*+:*MAT1-2−* ratio for the sum of the RAPD markers). It showed a value of 0.375 with 1 degree of freedom.

b The genotype of each progeny isolate, defined by unique combinations of mating-type and RAPD markers as distinct from those of the parental isolates (designated P1 and P2), is identified by a different letter of the alphabet.

In accordance with the “One Fungus = One Name” proposal (59), the newly discovered sexual state (teleomorph) of A. lentulus will not be assigned a separate Latin name, as was formerly the case under “dual nomenclature” (60). This follows the recent taxonomic move to simplify the naming of pleomorphic fungi. In the future, the teleomorph of A. lentulus should be referred to as A. lentulus (neosartorya-morph) where appropriate (59), given the phylogenetic link between the teleomorph genus Neosartorya and the Aspergillus section Fumigati (47, 56).

As shown in Table 1, three of the four fertile crosses (fertility is here defined as the production of cleistothecia with viable ascospores) yielded sexual offspring at both 28°C and 30°C, suggesting that A. lentulus may not be as fastidious as A. fumigatus in its temperature requirement for mating (61). Crosses were then reincubated for a further 4 weeks and reexamined for the presence of cleistothecia (see Table S3 in the supplemental material). The longer incubation period resulted in a further 7 and 14% of crosses reaching sexual maturity at 28°C and 30°C, respectively. Thus, a total of 35% of the crosses were fertile at both temperatures. A second series of 20 crosses was then set up at 28°C for 3 weeks to test the fertility of an additional seven isolates (see Table S4 in the supplemental material). Six of the seven isolates were fertile with at least one mating partner, although overall fertility was still only 35%. These results illustrate the importance of having multiple isolates of opposite mating type in close proximity in the environment, to ensure compatibility with at least one complementary strain.

Finally, crosses were attempted between representative *MAT1-1* and *MAT1-2* isolates of A. lentulus and highly fertile “supermater” isolates of A. fumigatus (37). Hyphal aggregations resembling immature cleistothecia were formed very occasionally in such crosses (see Fig. S3 in the supplemental material). However, despite prolonged incubation, for up to 12 months, at both 28°C and 30°C, these never matured to form ascospores. This indicates that the species are true sibling species, being phylogenetically closely related but without gene flow via sexual means (62). This has important implications for the evolution of resistance to antifungal drugs in A. fumigatus, as it suggests, fortunately, that transmission of genes conferring such resistance is unlikely to occur between A. lentulus and A. fumigatus. This result is consistent with the phylogenetic divergence reported between the species (2, 4), although it is cautioned that crosses were attempted only between a subset of A. lentulus and A. fumigatus isolates. It has also been reported that abortive cleistothecia can be produced in crosses between Neosartorya fennelliae and A. fumigatus (63). These crossing results compare with data for some other ascomycete species, such as within the genus Neurospora, where interspecies crossing is possible, albeit with reduced fertility and ascospore viability (64, 65).

The large variation in fertility of A. lentulus isolates depending on the mating partner is similar to observations reported for A. fumigatus by both O'Gorman et al. (25) and Sugui et al. (37) and for Neosartorya udagawae by Sugui et al. (66). For example, the A. lentulus cross 78-3 × 78-8 produced fewer than 20 cleistothecia per plate (Table 1). However, when 78-3 was crossed with 78-2 it was the most fertile pairing at both temperatures, consistently producing ≥80 cleistothecia per plate (Table 1). The pairing of 78-3 × 78-2 is therefore recommended for community use. It should be noted that these two isolates came from agricultural soils within 50 mi of each other in the Republic of Korea. Their similar geographic origins may hint at a close genetic relationship and lack of reproductive barriers, this being one of a variety of factors suggested to influence fertility of fungal sexual crosses (67–69). Genome relatedness is thought to be an important factor for fertility, as illustrated for A. fumigatus by the “supermater” pair (37). These isolates share 99% genome similarity based on a CGH (comparative genomic hybridization) analysis, and the cross is the most successful A. fumigatus pairing to date.

It is important to note that only 25% of the A. lentulus crosses at both 28°C and 30°C were fertile after 3 weeks. This figure is extremely low in comparison to that for A. fumigatus, for which the study by Sugui et al. (37) found that 80% (*n* = 50) of crosses were fertile after 4 weeks. O'Gorman et al. (25) reported 94% of their A. fumigatus crosses to be fertile (*n* = 36), but this was after 6 months of incubation, which can be considered to represent maximum maturity. The large disparity in fertility between A. lentulus and A. fumigatus is surprising given their nearly identical *MAT* idiomorph structures (Fig. 1) and conditions required for the sexual cycle. It is conceivable that either part or all of the global A. lentulus population is undergoing a slow decline in fertility (44). Alternatively, it could suggest a difference in the population dynamics of the two species due to an unknown underlying fertility or incompatibility mechanism in A. lentulus. Natural A. fumigatus populations have been well studied and have yielded evidence of recombination, confirming that sexual reproduction is taking or has recently taken place (35, 70, 71). Similar studies have yet to be conducted for A. lentulus, for which many fewer isolates are available for study. Defining its population structure will inform future studies to determine how widespread sexual reproduction is in nature and whether the low fertility seen in this study is representative of the global population.

### Conclusions.

The discovery of a sexual cycle in A. lentulus is important both for the biology of the species and for future efforts to control this pathogen. It also shows that yet another supposedly asexual pathogenic fungus possesses a functional sexual cycle, thereby harboring the potential to evolve rapidly in the face of selective pressures (43). Sexuality in its close relative A. fumigatus can now be directly compared to that of A. lentulus, and future studies may shed light on their different evolutionary paths. However, of most significance is the fact that the sexual cycle will provide an invaluable tool for classical genetic analysis to facilitate research into the genetic basis of pathogenicity and drug resistance in this emerging agent of aspergillosis.

## Supplementary Material

Supplemental material
