# Supplementary material for: Discovery of a Sexual Cycle in Aspergillus lentulus, a Close Relative of A. fumigatus
Source: Eukaryot Cell. 2013 Jul;12(7):962–9. doi: 10.1128/EC.00040-13 (PMC3697472; doi:10.1128/EC.00040-13)
Supplement: Supplemental material [file supp_12_7_962__index.html]

Supplemental material 

# Discovery of a Sexual Cycle in Aspergillus lentulus, a Close Relative of A. fumigatus

## 

**Files in this Data Supplement:**

- Supplemental file 1 -

  Fig. S1 to S3, Tables S1 to S4, and description of species diagnosis.

  PDF, 2.5M
