# Supplementary material for: Discovery of a Sexual Cycle in Aspergillus lentulus, a Close Relative of A. fumigatus
Source: Eukaryot Cell. 2013 Jul;12(7):962–9. doi: 10.1128/EC.00040-13 (PMC3697472; doi:10.1128/EC.00040-13)
Supplement: Supplemental material [file EC.00040-13_zek999094117so1.pdf]

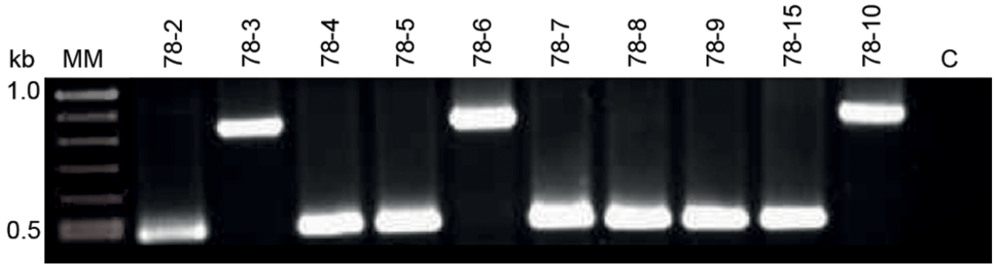

Fig. S1. Representative results from the multiplex mating-type PCR assay to determine the mating type of isolates of *A. lentulus*. Lanes 2-11 show amplicons of either ca. 834 bp (*MAT1-1*) or 438 bp (*MAT1-2*) from ten isolates (code numbers are indicated above each lane). MM, molecular weight marker; C, water control.

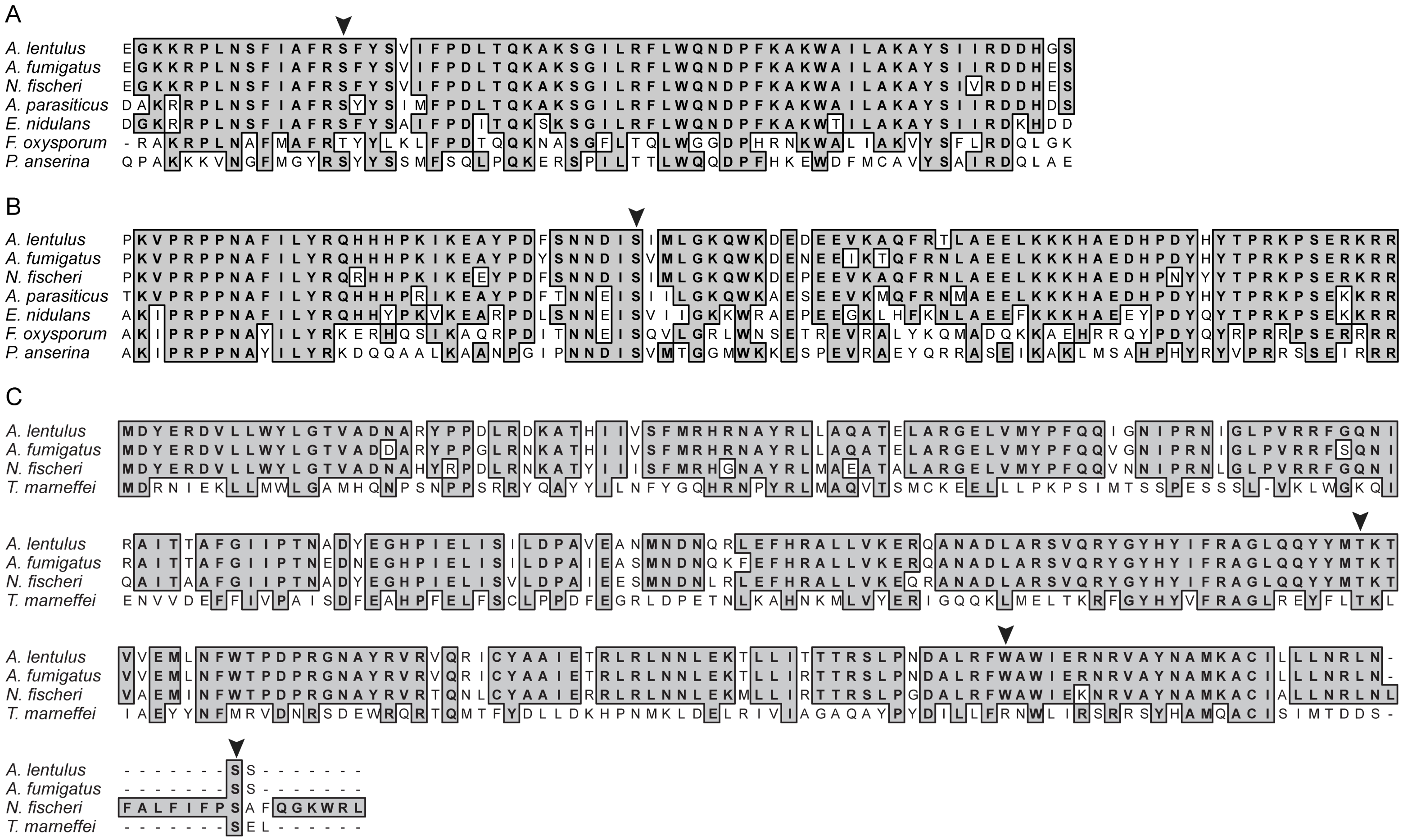

Fig. S2. Multiple alignments of predicted proteins from the *A. lentulus* sequences of the *MAT1-1-1* α1 domain, *MAT1-2-1* HMG domain, and *MAT1-2-4* ORF (the full sequence of *MAT1-2-4* is provided as there is no known domain present). Alignments show conservation with (A) α1 domain regions of MAT1-1 proteins, (B) HMG domain regions of MAT1-2 proteins, and (C) MAT1-2-4 translated ORFs, from other ascomycete fungi. Inverted arrows indicate locations of conserved introns. NCBI Accession numbers: *Aspergillus fumigatus* [EDP53120 (MAT1-1); XP_754989 (MAT1-2); XP_754990 (MAT1-2-4)], *Neosartorya fischeri* [XP_001263836 (MAT1-1); XP_001263957 (MAT1-2); XP_001263958 (MAT1-2-4)], *Aspergillus parasiticus* [ACA51903 (MAT1-1); ACA51906 (MAT1-2)], *Emericella nidulans* [ABC87272 (MAT1-1); AAQ07985 (MAT1-2)], *Fusarium oxysporum* [CAI45427 (MAT1-1); BAA28611 (MAT1-2)], *Podospora anserina* [P35692 (MAT1-1); P35693 (MAT1-2)], *Talaromyces marneffei* [XP_002488739 (MAT1-2-4)].

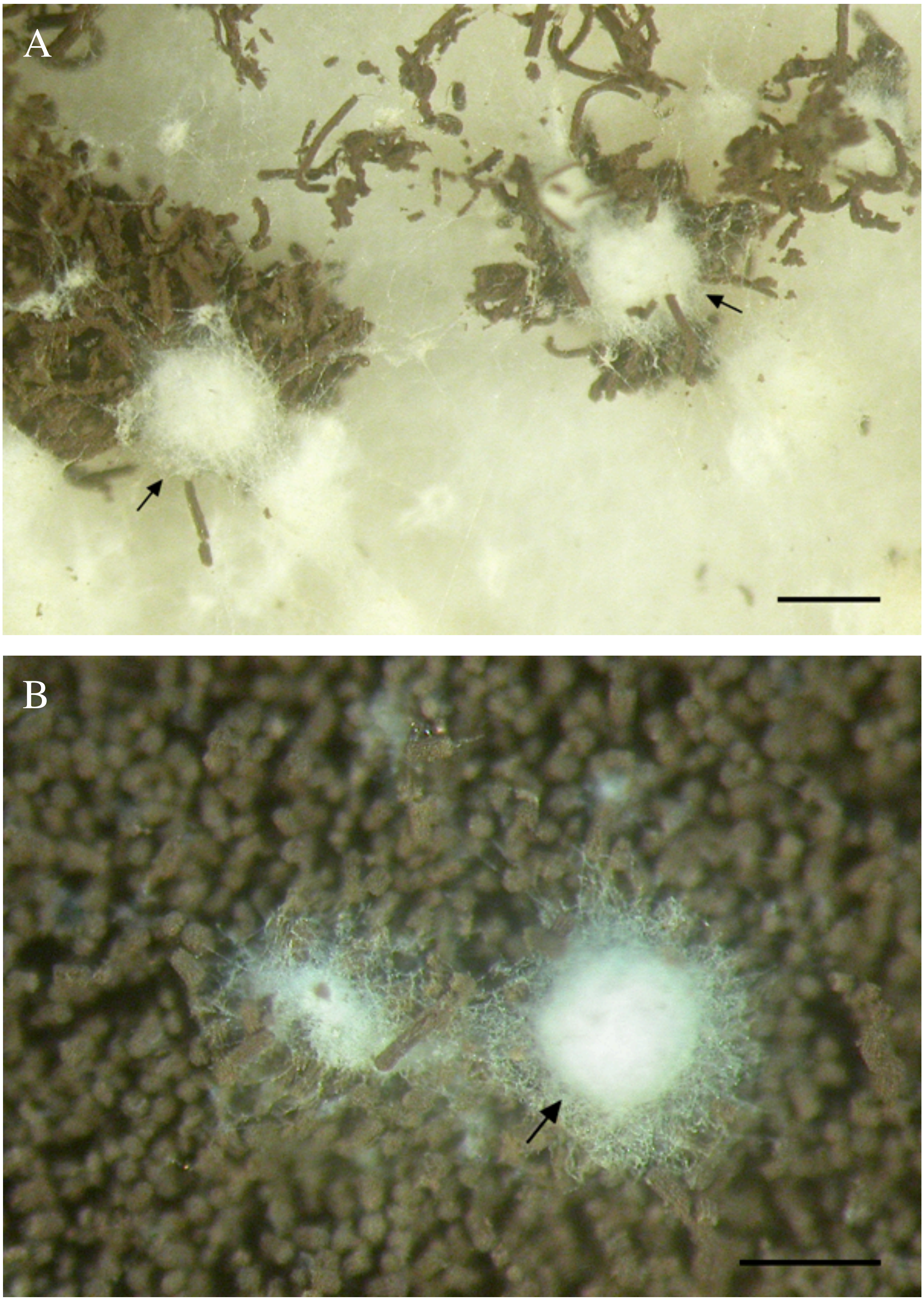

Fig. S3. Hyphal aggregates possibly representing abortive cleistothecia (arrowed) from two eight-month-old crosses between *A. fumigatus* strain AFB62 (*MAT1-1*) and *A. lentulus* strain 78-2 (*MAT1-2*). Scale bars, 150 μm.

Table S1. Details of *Aspergillus lentulus* isolates used in the study and their *MAT* locus genotype

BDUN number[a]	Alternative strain number(s)[b]	Source	Isolation site[c]	Country[c]	State/ County[c]	*MAT* Locus	Reference
78-1[d,e]	FH 5, CBS 117885, IBT 27201, KACC 41940, NRRL 35552	Clinical	Mouth	USA	Washington	*MAT1-2*	(2)
78-2[d]	CBS 116881, IBT 24948, KACC 41686	Environmental	Soil of maize field	Republic of Korea	Yeongi	*MAT1-2*	
78-3[d]	CBS 116882, KACC 41395	Environmental	Soil of pepper field	Republic of Korea	Chungju	*MAT1-1*	(1)
78-4[d]	CBS 116883, KACC 41393	Environmental	Soil of pepper field	Republic of Korea	Daejeon	*MAT1-2*	(1)
78-5[d]	CBS 117884, FH 4	Clinical	Lungs	USA	Washington	*MAT1-2*	(2)
78-6[d]	CBS 117886, FH 7	Clinical	Lungs	USA	Washington	*MAT1-1*	(2)
78-7[d]	IFI06-0001	Clinical	Lungs	USA	Washington	*MAT1-2*	(2)
78-8[d]	IFI06-0014	Clinical	Lungs	USA	Washington	*MAT1-2*	(2)
78-9[d]	IFI05-0046	Clinical	Lungs	USA	Washington	*MAT1-2*	(2)

78-10	CBS 117182, IBT 23163, IMI 306135	Environmental	Soil treated with herbicides	Australia	Western Australia	*MAT1-1*	
78-11	CBS 117180, IBT 23719, IMI 376377	Environmental	*Coffea* sp.	Denmark	N/A	*MAT1-1*	(6)
78-12	CBS 121597	Environmental	Cocoa beans	N/A	N/A	*MAT1-1*	
78-13[d]	CBS 175.97	Environmental	Dolphin nostril	Netherlands	Harderwijk	*MAT1-2*	(1)
78-14[d]	FH 1	Clinical	Lungs	USA	Washington	*MAT1-1*	(10)
78-15	FH 6	Clinical	Kidneys	USA	Washington	*MAT1-2*	(2)
78-16	FH 84	Clinical	N/A	USA	Georgia	*MAT1-1*	(20)
78-17	FH 85	Clinical	N/A	USA	Georgia	*MAT1-2*	(20)
78-18	FH 86	Clinical	N/A	USA	Georgia	*MAT1-2*	(20)
78-19[d]	FH 219	Clinical	Lungs	USA	Washington	*MAT1-2*	(5)
78-20[d]	CBS 117887, FH 220	Clinical	Mouth	USA	Washington	*MAT1-1*	(2)

78-21[d]	FH 231	Clinical	N/A	USA	Texas	*MAT1-2*	(20)
78-22	FH 238	Clinical	N/A	USA	Texas	*MAT1-1*	(20)
78-23	FH 239	Clinical	N/A	USA	Texas	*MAT1-1*	(20)
78-24[d]	FH 265	Clinical	N/A	USA	Washington	*MAT1-2*	(5)
78-25[d]	FH 292	Clinical	N/A	USA	Georgia	*MAT1-2*	
78-26	FH 293	Clinical	N/A	USA	Washington	*MAT1-2*	

[a] BDUN Culture Collection, School of Biology, University of Nottingham, UK.

[b] FH, Fred Hutchinson Cancer Research Center, WA, USA; CBS, Centraalbureau voor Schimmelcultures, Utrecht, The Netherlands; KACC, Korean Agricultural Culture Collection, Suwon, Republic of Korea; IBT, IBT Culture Collection of Fungi, Technical University of Denmark, Lyngby, Denmark; IMI, CABI Bioscience, Egham, UK; NRRL, Agricultural Research Service Culture Collection, US Department of Agriculture, IL, USA.

[c] N/A, Information not available.

[d] Used in sexual crossing experiments.

[e] Type strain.

Table S2. Primers used in the study

Primer	Sequence (5'–3')	Target region	Source or reference
AFM1	CCTTGACGCGATGGGGTGG	*MAT1-1* idiomorph	(36)
AFM2	CGCTCCTCATCAGAACAAC TCG	*MAT1-2* idiomorph	(36)
AFM3	CGGAAATCTGATGTCGCCAC G	'Common' flanking region	(36)
Loc1	CAGAAGGGGCAAAAGGAC AAGC	'Common' flanking region	(36)
AL31	CGGTTTCTCTCAATCCAAGC	*MAT1-2* idiomorph	This study
AL32	AGAAACATCGGCTTCAGGG	*MAT1-1* idiomorph	This study
AL33	TGGGCTTTGGTGTCTTGGAG	*MAT1-1* idiomorph	This study
AL34	GGTAACCGTAACGCTGAACG	*MAT1-2* idiomorph	This study
AL51	TAAGGCGGGATTGACACAC	*MAT1-1* idiomorph	This study
AL52	ATCTTCGGCGTGCTTCTTC	*MAT1-2* idiomorph	This study
AL53	AAGCATCCTGCCACAAGA	*MAT1-2* idiomorph	This study
OMT1	GTGGACGGACCTAGT CCGACATCAC	Unknown (RAPD)	(73)
R108	GTATTGCCCT	Unknown (RAPD)	(74)
R151	GCTGTAGTGT	Unknown (RAPD)	(74)
OPWO8	GACTGCCTCT	Unknown (RAPD)	RAPD 10mer kit W, Eurofins MWG Operon

Table S3. Mean numbers of cleistothecia* produced by 14 *Aspergillus lentulus* crosses on oatmeal agar at 28 °C or 30 °C in the dark after seven weeks

	Crosses	*MAT1-1*			
		28 °C		30 °C	
		78-3	78-6	78-3	78-6
MAT1-2	78-1	+	–	–	–
	78-2	>	+	++++	+++
	78-4	–	–	–	–
	78-5	–	–	–	–
	78-7	–	–	–	–
	78-8	+	+	+	–
	78-9	–	–	+	+

*Ratings indicate the mean number of cleistothecia produced from three replicate crosses on oatmeal agar in 9-cm diameter Petri dishes after incubation in the dark. –, none; +, 1-19; +++, 40-59; ++++, 80-100; >, more than 100 cleistothecia.

Table S4. Mean numbers of cleistothecia* produced by 20 *Aspergillus lentulus* crosses on oatmeal agar at 28 °C in the dark after three weeks

Crosses		*MAT1-1*			
		78-3	78-6	78-14	78-20
MAT1-2	78-13	+++	–	–	+
	78-19	–	–	–	++
	78-21	+	–	–	+
	78-24	+++	–	–	–
	78-25	–	–	–	++++

*Ratings indicate the mean number of cleistothecia produced from three replicate crosses on oatmeal agar in 9-cm diameter Petri dishes after incubation in the dark for three weeks at 28 °C: –, none; +, 1-19; ++, 20-39; +++, 40-59; ++++, 80-100; >, more than 100 cleistothecia.

Diagnosis of the *Aspergillus lentulus* neosartorya-morph

MycoBank No.: MB356679

Heterothallic fungus. Cleistothecia produced on oatmeal agar at 28 °C and 30 °C after three weeks, superficial, globose to subglobose, 300-500 μm in diameter, grouped in clusters, at first white then turning light yellow to pale orange-yellow. Peridium composed of several layers of tightly interwoven, often flattened hyphae. Asci globose to subglobose, hyaline, containing eight ascospores, evanescent at maturity. Ascospores spherical to lenticular, 3-4 x 4-6 μm, with two distinct equatorial ridges, often with additional inner partial secondary ridges, convex surface ornamention of reticulate ridges and small point projections. Anamorph: *Aspergillus lentulus* Balajee & K.A. Marr 2005.

Holotype: Dried oatmeal agar specimen with teleomorph of paired *Aspergillus lentulus* colonies, 78-6 (*MAT1-1*) x 78-2 (*MAT1-2*), deposited with the Fungarium of the Royal Botanic Gardens, Kew (K) [K(M) 181782].

Supplementary References

73. **Geisen R.** 1996. Multiplex polymerase chain reaction for the detection of potential aflatoxin and sterigmatocystin producing fungi. Syst. Appl. Microbiol. **19:**388–392.

74. **Aufauvre-Brown A, Cohen J, Holden DW.** 1992. Use of randomly amplified polymorphic DNA markers to distinguish isolates of *Aspergillus fumigatus*. J. Clin. Microbiol. **30:**2991–2993.
